# Reverse Correlation between MicroRNA-145 and FSCN1 Affecting Gastric Cancer Migration and Invasion

**DOI:** 10.1371/journal.pone.0126890

**Published:** 2015-05-26

**Authors:** Jia-jia Chen, Wang-yu Cai, Xue-wen Liu, Qi-cong Luo, Gang Chen, Wei-feng Huang, Na Li, Jian-chun Cai

**Affiliations:** 1 Department of Gastrointestinal Surgery, Zhongshan Hospital of Xiamen University, Xiamen, Fujian, China; 2 Institute of Gastrointestinal Oncology, Medical College of Xiamen University, Xiamen, Fujian, China; Michigan State University, UNITED STATES

## Abstract

MicroRNAs (miRs) play important roles in modulating gene expression during the processes of tumorigenesis and tumor development. Previous studies have found that miR-145 is down-regulated in the stomach neoplasm and is related to tumor migration and invasion. However, both the molecular mechanism and function of miR-145 in gastric cancer remain unclear. The present study is the first demonstration of the significant down-regulation of miR-145 expression in infiltrative gastric cancer compared to expanding gastric cancer. Additionally, correlation analyses revealed strong inverse correlations between miR-145 and FSCN1 expression levels in infiltrative gastric cancer. Furthermore, we demonstrated that miR-145 directly targets FSCN1 and suppresses cell migration and invasion in gastric cancer. Knocking down the expression of FSCN1 led to the suppression of migration and invasion in gastric cancer cells, and re-expressing FSCN1 in miR-145-overexpressing cells reversed their migration and invasion defects. Thus, we concluded that miR-145 regulates cell migration and invasion in gastric cancer primarily by directly targeting FSCN1.

## Introduction

Gastric cancer has one of the highest death rates for malignant tumors worldwide [1]. Improved medical technology may improve the outcome of gastric cancer. However, gastric cancer remains the second most common cause of cancer-related deaths [2], which is caused by tumor invasion and metastasis. Together, invasion and metastasis form a process in which malignant tumor cells transfer from a primary site to other areas and then form tumors at a distant site through the lymphatic channel, blood vessels or body cavity [3]. Hence, clarifying the molecular mechanisms involved in the development and invasiveness of gastric cancer is necessary.

In 1977, Ming classified gastric carcinomas into expanding and infiltrative types based on their growth and invasiveness patterns [4]. These types are readily recognizable histologically. Expanding carcinomas grow en masse and by expansion resulting in the formation of discrete tumor nodules, whereas infiltrative carcinomas have tumor cells that invade individually [5]. Of the two types of gastric cancer, infiltrative gastric cancer is more invasive than expanding gastric cancer. Such a classification thus provides a reference to distinguish gastric cancer samples clinically and facilitates detailed studies regarding the molecular mechanisms of gastric cancer.

MicroRNAs (miRs) play critical roles in many biological processes, including cancer processes, by directly inhibiting the expression of target mRNAs through various molecular mechanisms [6, 7]. Additionally, miRs undergo aberrant regulation during carcinogenesis and can act as either oncogenes or tumor suppressors [7]. Recent studies have shown that many miRs affect proliferation and invasion in gastric cancer. miR-21 is up-regulated in gastric cancer and promotes tumor proliferation and invasion in gastric cancer by targeting PTEN [8]. In contrast, miR-145 is down-regulated in human gastric cancer [9]. Moreover, research has shown that miR-145 can suppress cell migration and invasion by inhibiting N-cadherin protein translation in gastric cancer cells [10]. However, Kamikihara et al [11] detected the expression levels of N-cadherin in 146 patients with gastric cancer by immunohistochemistry, and the results showed that only 31 patients (21%) had N-cadherin-positive expression. Therefore, some other molecular mechanisms of miR-145 might regulate cell migration and invasion in gastric cancer.

FSCN1 is a 55-kDa actin-binding protein that is associated with filopodia and actin-based protrusion formation, and it promotes cell motility, migration and invasion [12, 13]. FSCN1 expression is absent or low in normal epithelia, but FSCN1 is overexpressed in many carcinomas, including colorectal adenomas, epithelial ovarian cancer and esophageal squamous cell carcinoma [14–16]. Moreover, a series of studies have indicated that enforced FSCN1 expression increases the proliferation and invasion of ovarian and gastric cancer cells [17, 18].

Recent studies have found that miR-145 functions as a tumor suppressor and that miR-145 overexpression suppresses migration and invasion in prostate cancer and bladder cancer by targeting FSCN1 [19, 20]. In addition, miR-145 suppresses gastric cancer cell migration and invasion by targeting N-cadherin. However, the positive expression rate of N-cadherin in gastric cancer tissues is only 21% [10, 11]. Therefore, determining the key target gene of miR-145 in regulating gastric cancer invasion is necessary.

In the present study, we first found that miR-145 expression was remarkably down-regulated in infiltrative gastric cancer compared to that in expanding gastric cancer. Moreover, we provided evidence that miR-145 suppressed cell migration and invasion in gastric cancer primarily by directly targeting FSCN1, which highlighted the role of miR-145 and FSCN1 in the regulation of gastric cancer malignant phenotypes.

## Materials and Methods

### Clinical samples

All clinical samples were collected with the informed consent of patients who underwent gastrectomy at the Zhongshan Hospital of Xiamen University in Xiamen (Fujian Province, China) from June 2012 to October 2013. In total, the 160 pairs of collected clinical samples included 80 unclassified gastric cancer samples, 40 infiltrative gastric cancer samples and 40 expanding gastric cancer samples. The tumor pathological type was confirmed by pathology examination, and the matched normal gastric mucosae were collected from more than 5 cm away from the tumors.

### Ethics statement

The study protocols were in accordance with the ethical guidelines of the Declaration of Helsinki (1975) and were approved by the Medical Ethics Committee (No. 20081012) of Zhongshan Hospital of Xiamen University in Xiamen (Fujian Province, China). We obtained written consent statements from all participants involved in this study.

### Cell lines

The MGC-803 and SGC-7901 human gastric cancer cell lines were purchased from the Institute of Cell Biology (Shanghai, China, http://www.cellbank.org.cn). The 293T human embryonic kidney cell line was obtained from the American Type Culture Collection (ATCC, MD, USA). MGC-803 and SGC-7901 cells were maintained in RPMI-1640 medium, while 293T cells were maintained in DMEM. All media contained 10% fetal bovine serum (FBS), 100 U/ml penicillin and 100 μg/ml streptomycin (Invitrogen, Carlsbad, CA, USA).

### Luciferase reporter assay

Cells were transfected with luciferase reporter genes, β-galactosidase (β-gal) and miR-145 mimics or miR-145 inhibitor (Guangzhou RiboBio) using Lipofectamine 2000 transfection reagent (Invitrogen). Luciferase activity was measured at 48 h after transfection, and the transfection efficiency was normalized to internal β-gal activity.

### RNA extraction and RT-qPCR

Total RNA was extracted using TRIzol reagent (Invitrogen) and reverse-transcribed with M-MuLV reverse transcriptase (Qiagen) to produce cDNA according to the manufacturer’s protocol. Real-time PCR was performed using SYBR Green-based detection in a Rotor-Gene 6000 instrument (Corbett Life Science) according to the manufacturer’s instructions and using the primer pairs listed in Table 1. miR-145 and U6 primers were obtained from Qiagen. GAPDH or U6 endogenous control was used as an internal standard, and the results were calculated using the △△CT (where CT is the threshold cycle) method.

**Table 1:** Primer sequences used in this study.

Name	Sequence (from 5’→3’)
FSCN1 qPCR	Forward: TCAGAGCTCTTCCTCATGAAGCT
	Reverse: GTCCAGTATTTGCCTGTGGAGTC
GAPDH qPCR	Forward: TCTCCTCTGACTTCAACAGCGA
	Reverse: GTCCACCACCCTGTTGCTGT
shFSCN1-1	Target sequence: CCCTTGCCTTTCAAACTGGAA
shFSCN1-2	Target sequence: CAAGTTTGTGACCTCCAAGAA
WT-FSCN1 3’ UTR	Forward: CCCGAGCTCCCTTGCCTTTCAAACTGGAAA
	Reverse: TGCACGCGTGGGGCTGCAGACTGAGTTATTT
Mut-FSCN1 3’ UTR	Forward: CTTGCCTTTCAAAGTCCAAACCCCAGAGA
	Reverse: GGACTTTGAAAGGCAAGGGGGCTTGCC
	Forward: GGGCGTGTAGTGTAAGTCCAATCTTTTGCCT
	Reverse: GGACTTACACTACACGCCCAGGGCTCCCAG
	Forward: ATAGTAGCTTCAAAGTCCAAATAGCGAAATA
	Reverse: GGACTTTGAAGCTACTATCATGGGCGTTTA
miR-145-expressed sequence	Forward: TCCACTAGTCAGAGGGTTTCCGGTACTTTTCA
	Reverse: TCGGCTAGCGATGGAAAGAAAAGCAACGCAA
FSCN1-expressed sequence	Forward: TCATCTAGACGGCCTCTCGTCTACTGCCA
	Reverse: TACCATATGCTAGTACTCCCAGAGCGAGGCG

### Plasmid construction

The primers used for the construction of the luciferase reporters and plasmids are listed in Table 1. The human FSCN1 3’ untranslated region (UTR) was amplified from MGC-803 cDNA by PCR amplification with the LA Taq DNA Polymerase (TaKaRa) and cloned downstream of the luciferase coding sequence in the pMIR-REPORT (Ambion) vector at the SacI and MluI restriction sites (Promega) to construct the human FSCN1-3’UTR-luciferase reporter. Mutations were introduced into the miR-binding sites using a QuikChange Mutagenesis Kit (TransGen). For the miR-145 and FSCN1 expression plasmids, sequences were amplified by PCR and cloned into the SpeI/NheI sites and XbaI/NdeI sites, respectively, of the Plv-cs.4.0-basic vector (Promega). For the FSCN1 interference plasmid, two pairs of sequences were annealed and subcloned into PLKO.1 according to the manufacturer’s protocol.

### Lentiviral production and transfection

HEK293T cells were seeded on a 6-well insert at 24 h before transfection. The miR-145 expression plasmid, FSCN1 shRNA plasmid or FSCN1 expression plasmid was then co-transfected with packaging plasmids (pHR and pCMV-VSV-G) using the Lipofectamine 2000 transfection reagent (Invitrogen). Viral supernatants were collected at 48 h after transfection, centrifuged at 3000 g for 15 min, and filtered through 0.45 μm filters (Millipore). Viral titers were determined by transducing HeLa cells at serial dilutions and analyzing GFP expression using flow cytometry. MGC-803 cells or 7901 cells at 50–70% confluency were infected with viral supernatants containing 10 mg/ml Polybrene for 24 h. Fresh medium was then added to the infected cells, which were later selected with puromycin.

### Cell migration and invasion assay

Cells (3x10^5^) were plated on 8.0 μm pore size Boyden chambers (Transwell, Corning Life Sciences, Acton, MA, USA) either coated with 10 μg of Matrigel (BD Bioscience, Bedford, MA, USA) per well (for invasion assays) or left uncoated (for migration assays) in serum-free medium containing 10 g/L bovine serum albumin. Medium containing 10% FBS served as a chemoattractant in the lower chamber. Non-invading cells were removed with cotton swabs after 36 h (for invasion assays) or 20 h (for migration assays). Invaded cells were stained with hematoxylin (HE) and counted. The data are presented as the mean±SD from three individual experiments.

### Western blotting

Proteins were extracted with erythrocyte lysis buffer (ELB; 50 mM Tris, 140 mM NaCl, 0.5% NP-40 and 100 mM NaF (pH 7.6)) containing 1 mM phenylmethanesulfonyl fluoride (PMSF; Sigma–Aldrich, USA). Each sample was separated on a 10% SDS-PAGE gel and then transferred onto polyvinylidene difluoride (PVDF) membranes. The membranes were blocked with 5% milk and incubated with primary rabbit anti-human FSCN1 antibody (1:3000; Cell Signaling Technology, USA) or mouse anti-human GAPDH (1:5000; Sigma–Aldrich) antibody overnight at 4°C. The membranes were then incubated with the appropriate horseradish peroxidase (HRP)-conjugated secondary antibodies (Thermo Fisher Scientific). The bands were developed using an enhanced chemiluminescence (ECL) system (Amersham Biosciences, Buckinghamshire, UK). The GAPDH protein was used as a loading control.

### Statistical analysis

The data are expressed as the mean±SD. Statistical significance was analyzed with Student’s t-test. The correlation between the miR-145 levels and FSCN1 mRNA/protein expression levels in human gastric cancers was calculated by Spearman’s correlation. All statistical analyses were calculated using GraphPad Prism statistics software (GraphPad Software). *P*<0.05 was considered statistically significant (NS, not significant; *P<0.05; **P< 0.01; *** *P*<0.001).

## Results

### Strong inverse relation between the expression of miR-145 and its putative target, FSCN1, in human infiltrative type gastric cancer

We used the TargetScan database (http://www.targetscan.org) to identify the 3’ UTRs of specific target mRNAs of miR-145 that may regulate the migration and invasion of gastric cancer cells. Of all predicted mRNAs, we selected FSCN1, which has been previously implicated in tumor migration and invasion. FSCN1 also has highly conserved sequences at the 3’ UTR that are complementary to the miR-145 seed sequence.

**Fig 1:**
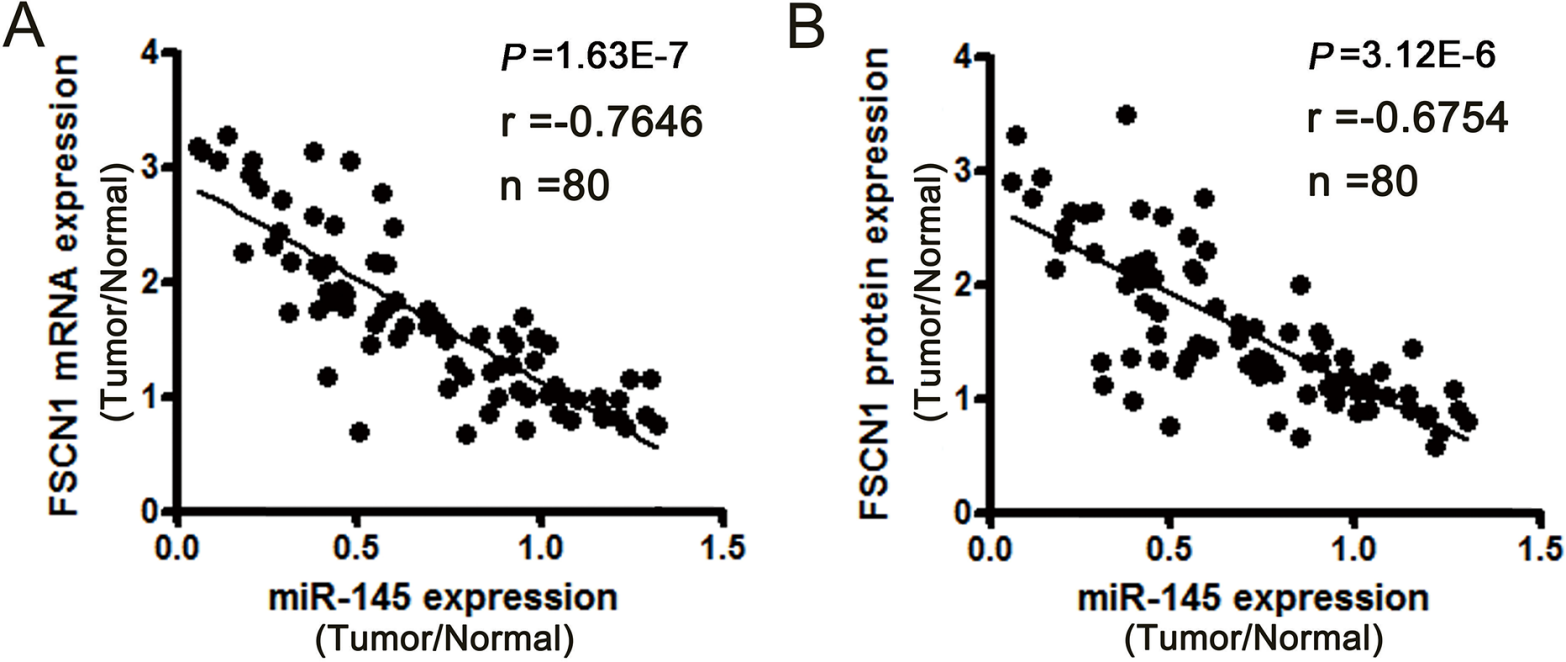
Relative expression of miR-145 and its potential target, FSCN1, in normal/tumor paired samples from patients with gastric cancer. (A) Inverse correlation between miR-145 expression and FSCN1 mRNA levels in normal/tumor paired gastric cancer samples (*P* = 1.63E-7; Spearman rank correlation test: r = -0.7646; n = 80). (B) Inverse relation between miR-145 expression and FSCN1 protein levels (*P* = 3.12E-6; r = -0.6754; n = 80).

First, we investigated whether the correlation of miR-145 and FSCN1 could be clinically relevant by measuring the expression of miR-145 and FSCN1 mRNA/protein levels in normal/tumor paired samples from 80 patients with gastric cancer. The statistical analysis revealed a significant inverse correlation between the expression of miR-145 and the expression of FSCN1 mRNA/protein in the 80 pairs of clinical samples (Fig 1A and 1B).

**Fig 2:**
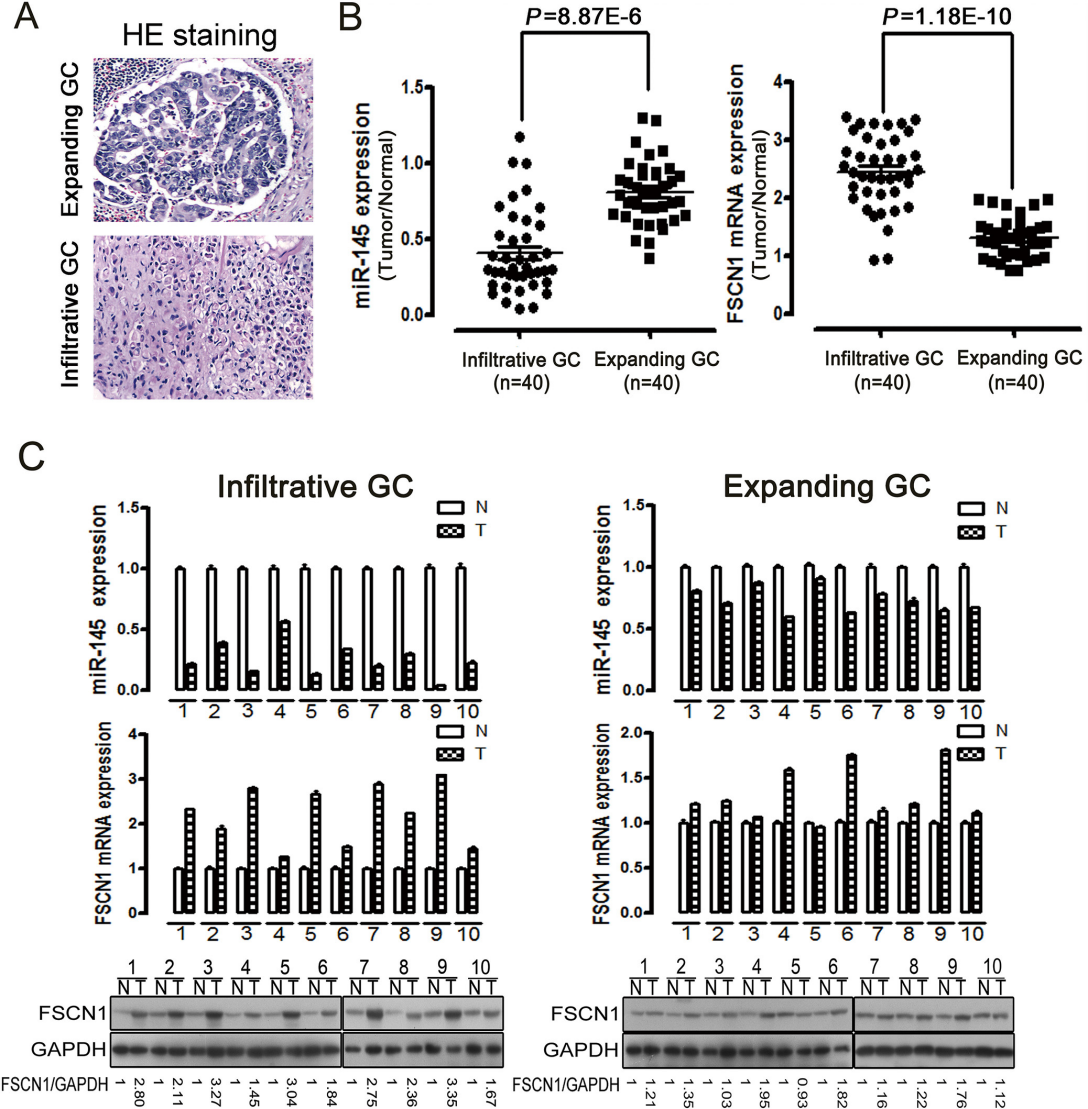
Strong inverse relation between miR-145 expression and FSCN1 expression in human infiltrative type gastric cancer. (A) Gastric cancer samples derived from 80 patients were classified as indicated by the representative HE staining. (B) qPCR data of miR-145 levels in infiltrative gastric cancer (n = 40) compared to expanding gastric cancer (n = 40) (*P* = 8.87E-6) (left), and qPCR data of FSCN1 mRNA levels in infiltrative gastric cancer (n = 40) compared to expanding gastric cancer (n = 40) (*P* = 1.18E-10) (right). (C) Representative expression of miR-145 and FSCN1 mRNA/protein levels in 10 infiltrative gastric cancer samples and 10 expanding gastric cancer samples along with their adjacent normal tissues (N, adjacent normal tissue; T, tumor tissue). Arabic numbers indicate individual sample pairs. The data are presented as the mean±SD of three independent experiments.

Ming classified gastric carcinomas into expanding type and infiltrative type based on growth and invasiveness patterns, and a correlation exists between Ming’s classification and the clinical prognosis [5]. Therefore, 40 infiltrative gastric cancer samples and 40 expanding gastric cancer samples were chosen as indicated by the representative HE staining (Fig 2A). In this classification, the cells of an expanding carcinoma, by virtue of their limited penetrating power, aggregate and produce a circumscribed mass in the form of polypoid and fungating lesions. In contrast, the cells of infiltrative carcinoma spread peripherally and widely, and a tumor mass does not form. We tested the expression of miR-145 and FSCN1 mRNA in gastric cancer compared to the adjacent normal gastric mucosa by real-time PCR. miR-145 was remarkably down-regulated in infiltrative gastric cancer compared to expanding gastric cancer, while the expression of FSCN1 mRNA was remarkably up-regulated (Fig 2B). We selected 10 infiltrative gastric cancer samples and 10 expanding gastric cancer samples along with their adjacent normal tissues to analyze expression by qPCR and western blotting. The overall expression levels of miR-145 were lower, particularly in the infiltrative gastric cancer samples compared to the adjacent normal gastric mucosa. In contrast, the overall mRNA/protein levels of FSCN1 were higher in the infiltrative gastric cancer (Fig 2C). Consequently, the expression of miR-145 and its putative target FSCN1 had a strong inverse relation in human infiltrative type gastric cancer, thereby suggesting that the essential roles of miR-145 and FSCN1 are in gastric cancer invasion.

### miR-145 directly targets FSCN1 in gastric cancer cells

**Fig 3:**
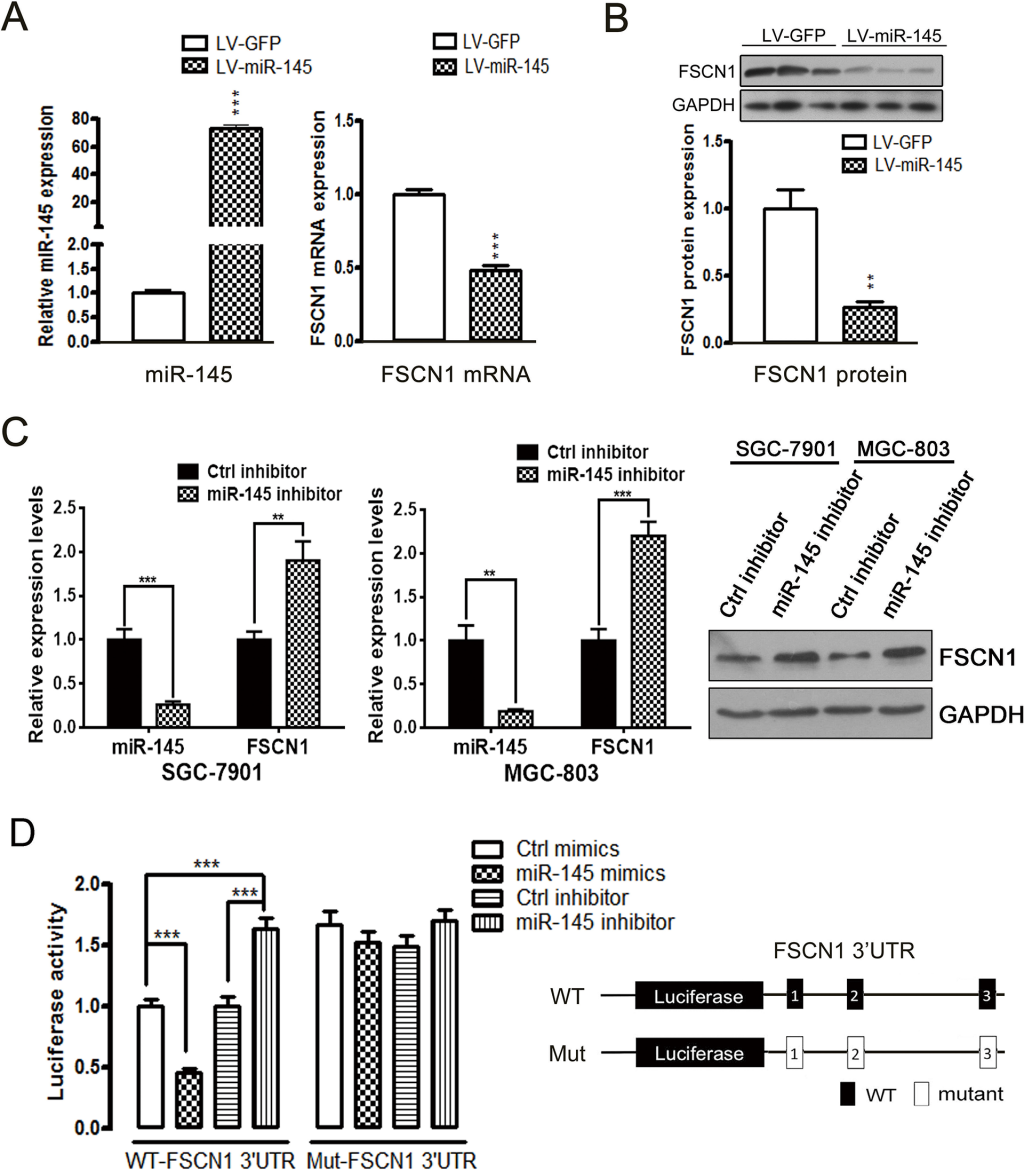
miR-145 suppresses FSCN1 expression by directly targeting the 3’ UTR of FSCN1. (A) qPCR data of FSCN1 mRNA levels in MGC-803 cells stably expressing the LV-GFP (control) and LV-miR-145 lentiviral vectors (right), and qPCR results showing miR-145 overexpression levels (left). (B) miR-145 overexpression significantly down-regulated the expression of the FSCN1 protein in MGC-803 cells. (C) qPCR data of miR-145 levels and FSCN1 mRNA levels in SGC-7901 (left) or MGC-803 (middle) cells transfected with Ctrl inhibitor or miR-145 inhibitor. The miR-145 inhibitor significantly up-regulated the expression of the FSCN1 protein in SGC-7901 and MGC-803 cells (right). (D) A luciferase reporter vector containing human WT-FSCN1 3’ UTR or Mut-FSCN1 3’ UTR was co-transfected with miR-145 mimics or miR-145 inhibitor into MGC-803 cells. Luciferase activity was measured at 48 h after transfection and normalized to β-gal activity. The data are presented as the mean±SD of three individual experiments (**P<0.01; ***P<0.001).

To determine if miR-145 regulates the expression of FSCN1 in gastric cancer cells, we first up-regulated the level of miR-145 in gastric cancer MGC-803 cells using a lentivirus-based vector. We then performed qPCR, which revealed that miR-145 overexpression significantly down-regulated the mRNA level of FSCN1 (Fig 3A), and western blot experiments confirmed that the protein level of FSCN1 was also suppressed in miR-145-overexpressing cells (Fig 3B). Conversely, knockdown of miR-145 levels using a miR-145 inhibitor resulted in significantly induced FSCN1 mRNA/protein expression in both SGC-7901 and MGC-803 cells (Fig 3C). We generated a firefly luciferase reporter vector containing the FSCN1 3’ UTR and then co-transfected MGC-803 cells with miR-145 mimics or a miR-145 inhibitor to investigate if FSCN1 is a direct target of miR-145. The lysates were analyzed for luciferase activity 24 h post-transfection. The luciferase assay showed that the miR-145 mimics resulted in an approximate 55% decrease in activity, whereas the miR-145 inhibitor increased the luciferase activity by 62% in MGC-803 cells compared with the miR-negative control (Fig 3D). Moreover, we mutated three major putative miR-145 recognition elements (MREs) in the FSCN1 3’ UTR via QuikChange mutagenesis for use in the luciferase assay (Fig 3D). The results showed that the regulatory effects of the miR-145 mimics or miR-145 inhibitor were primarily abrogated, which confirmed that the silencing effects of miR-145 on FSCN1 were abolished by the disruption of the miR–MRE interactions (Fig 3D). Collectively, these studies provided compelling evidence that FSCN1 is a direct target of miR-145 in gastric cancer cells.

### Repression of FSCN1 is necessary for miR-145 to inhibit the migration and invasion of gastric cancer cells

**Fig 4:**
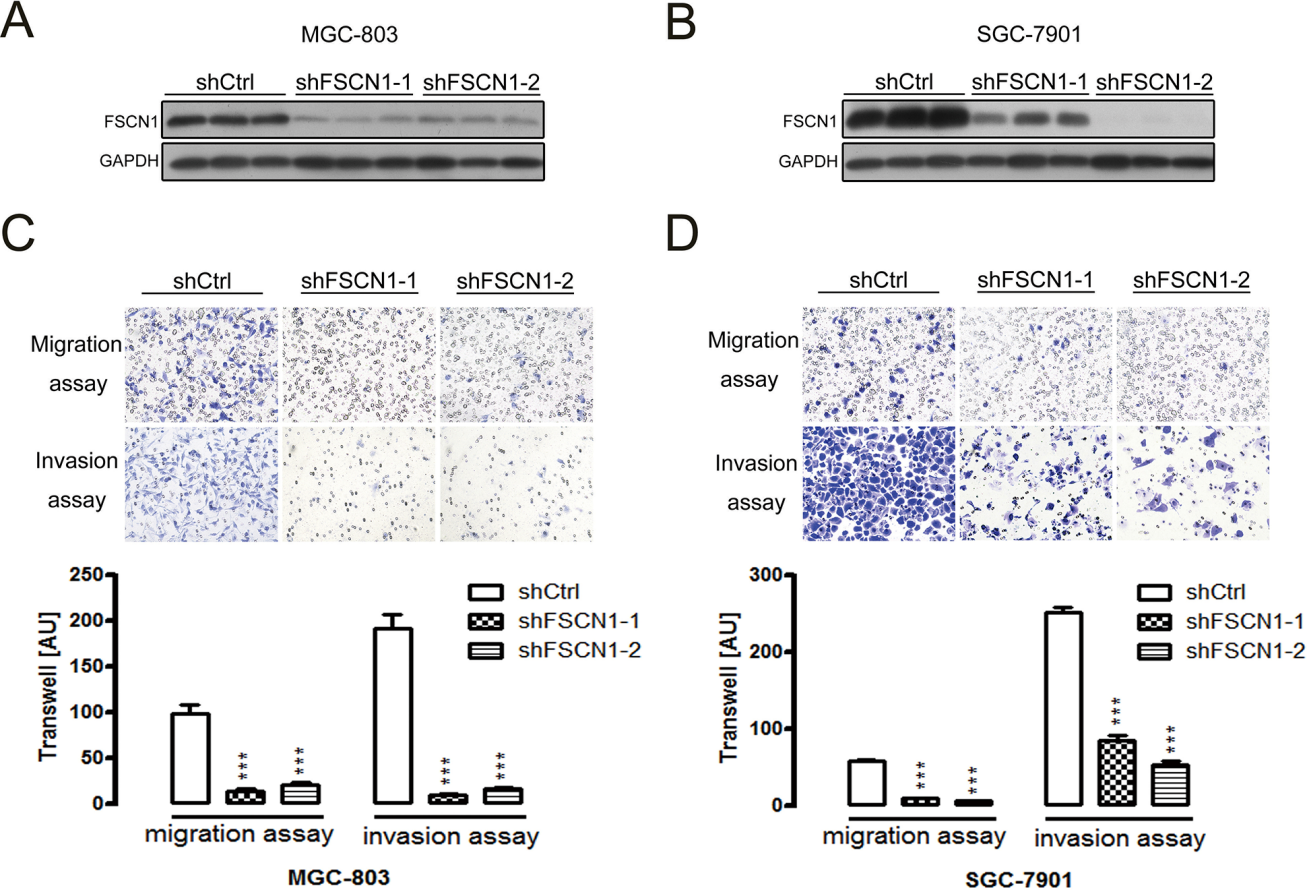
Migration and invasion efficiencies of gastric cancer cell lines stably expressing lacZ shRNA (control) and two different FSCN1 shRNAs. (A and B) Western blotting was used to measure the relative expression of FSCN1 protein in MGC-803 (A) and SGC-7901 (B) cells stably expressing lacZ shRNA and two different FSCN1 shRNAs. (C and D) ShFSCN1 MGC-803 (C) or SGC-7901 (D) cells exhibited decreased migration and invasion ability compared to shCtrl MGC-803 or SGC-7901 cells. Representative images (top) and cell counts (bottom) are shown. AU: arbitrary unit. The data are presented as the mean±SD of three individual experiments (***P<0.001).

Gastric cancer MGC-803 and SGC-7901 cells were stably transfected with two different FSCN1 shRNAs to inhibit FSCN1 to better understand the potential role of FSCN1 in miR-145–mediated tumor migration and invasion. The interference efficiency of FSCN1 shRNAs was confirmed by western blot analysis (Fig 4A and 4B). The transwell assay results showed that the ability of cells to migrate and invade was highly suppressed after knocking down the expression of FSCN1 in MGC-803 and SGC-7901 cells compared to negative control cells (Fig 4C and 4D), thereby suggesting an essential role of FSCN1 in these processes.

**Fig 5:**
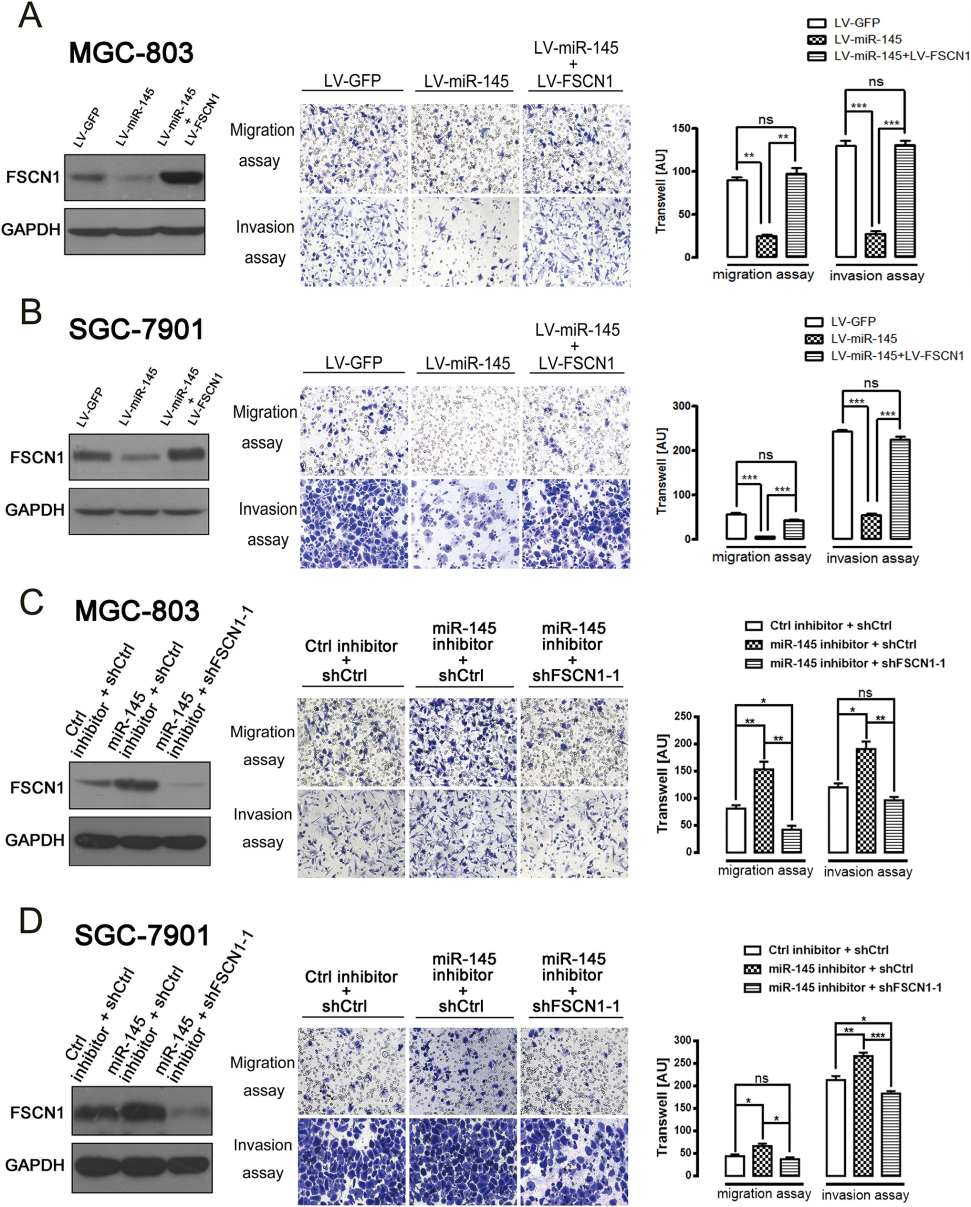
Repression of FSCN1 is necessary for miR-145 to inhibit the migration and invasion of gastric cancer cells. (A and B) Re-expression of FSCN1 compromised the miR-145-supressed cell migration and invasion ability in both miR-145-transfected MGC-803 (A) and SGC-7901 (B) cells. FSCN1 protein levels (left), representative images (middle) and cell counts (right) are shown. (C and D) Knockdown of FSCN1 compromised the miR-145 inhibitor-induced cell migration and invasion ability in both miR-145 inhibitor-transfected MGC-803 (C) and SGC-7901 (D) cells. FSCN1 protein levels (left), representative images (middle) and cell counts (right) are shown. AU: arbitrary unit. The data are presented as the mean±SD of three individual experiments (ns, not significant; *P<0.05; **P<0.01; ***P<0.001).

miR-145 is known to suppress the migration and invasion of many cancer types, including gastric cancer [10, 19, 21]. As expected, enforced expression of miR-145 resulted in significantly decreased migration and invasion rates in MGC-803 and SGC-7901 cells. Furthermore, simultaneous re-expression of FSCN1 compromised the miR-145-supressed cell migration and invasion ability almost completely in both miR-145-transfected MGC-803 and SGC-7901 cells (Fig 5A and 5B). Conversely, we used FSCN1 shRNA to inhibit FSCN1 expression in miR-145 inhibitor-transfected MGC-803 and SGC-7901 cells. Knockdown of FSCN1 completely reversed the miR-145 inhibitor-mediated activation of cell migration and invasion, thus suggesting the essential role of FSCN1 in this process (Fig 5C and 5D). Collectively, the above results demonstrated that the repression of FSCN1 is necessary for miR-145 to inhibit the migration and invasion of gastric cancer cells.

## Discussion

The deregulation of miRs has been implicated in various human cancers, including human gastric cancer. However, the molecular mechanisms by which miRs modulate the process of carcinogenesis and the behavior of cancer cells have not been completely defined. Differential miR expression in tumors compared with their adjacent normal tissues or between groups of tumor samples with a favorable or poor clinical outcome has been used to generate miR signatures with potential prognostic and/or predictive value in certain cancer types. Nevertheless, the involvement of the aberrantly expressed miRs in human gastric cancer remains largely unexplored. Previous studies have reported that miR-145 is down-regulated in various human malignancies, including breast cancer, lung cancer and gastric cancer [9, 22, 23]. Lu et al [24] found that miR-145 functions as a tumor suppressor and targets two oncogenes, namely ANGPT2 and NEDD9, in renal cell carcinoma. Another study has shown that miR-145 suppresses cell migration and invasion by inhibiting N-cadherin protein translation in gastric cancer cells [10]. However, Kamikihara et al [11] found that only 21% gastric cancer patients have N-cadherin-positive expression as analyzed by immunohistochemistry. Therefore, an alternative molecular mechanism underlying miR-145 is involved in the regulation of cell migration and invasion of gastric cancer.

In the present study, we confirmed that miR-145 is down-regulated in gastric cancer tissues compared to the adjacent normal gastric mucosa, which is consistent with previous findings [9]. In an attempt to identify novel downstream targets of miR-145, we used the TargetScan database to perform screening experiments. From several candidates, we selected FSCN1 for further experiments because FSCN1 has been found to play important roles in tumor migration and invasion. Several lines of evidence suggest that FSCN1 is the direct downstream target of miR-145. Firstly, FSCN1 contains highly conserved sequences at the 3’ UTR that are complementary to the miR-145 seed sequence. Secondly, an inverse correlation between miR-145 and FSCN1 was found in the paired samples from gastric cancer patients. Finally, when classifying the clinical samples into expanding type and infiltrative type based on Ming’s classification, we found that miR-145 is remarkably down-regulated in infiltrative gastric cancer compared to expanding gastric cancer. Meanwhile, a stronger inverse correlation was also detected between miR-145 and FSCN1 expression levels in infiltrative gastric cancer. The present study shed new light on the correlation between miR-145 and FSCN1 in infiltrative gastric cancer. In a previous study, Lauren divided gastric cancers into the following two types: intestinal type and diffuse type [25]. Intestinal type cancer is described as a glandular tumor resembling colonic carcinoma, and diffuse type cancer is composed of solitary or small clusters of cells without the formation of glands. The tumor types in Lauren's and Ming’s classification closely correspond to each other. Most expanding tumors have features of intestinal-type cancer, and most infiltrative tumors are diffuse type cancers [5]. However, a significant number of gastric carcinomas cannot be classified into intestinal type or diffuse type, including undifferentiated cancers that do not infiltrate diffusely and glandular cancers that infiltrate diffusely. In addition, the detection of early gastric cancer using Lauren's classification is relatively difficult because the early stage of diffuse type cancer, by definition, is not diffuse. However, Ming’s classification may solve these problems [5]. In Ming’s classification, the patterns of cell distribution clearly indicate a fundamental difference in their growth potential as well as power of penetration and invasion. As infiltrative gastric cancer is more invasive than expanding gastric cancer, these results suggest that miR-145 and FSCN1 both play crucial roles in gastric cancer invasion.

Although several reports have implicated the regulation of FSCN1 by miRs in many carcinomas [19, 20, 26], to the best of our knowledge, no report has suggested that FSCN1 is a direct target of miR-145 in gastric cancer. In this study, we confirmed that miR-145 represses FSCN1 expression and directly targets the 3’ UTR of FSCN1 in gastric cancer cells. In addition, the simultaneous re-expression of FSCN1 compromised the miR-145-supressed cell migration and invasion ability almost completely in miR-145-transfected MGC-803 and SGC-7901 cells, which indicated that the repression of FSCN1 is necessary for miR-145 to inhibit the migration and invasion of gastric cancer cells. Therefore, FSCN1 is an important downstream target of miR-145 in regulating gastric cancer invasion.

In summary, the present study identified that miR-145 is primarily down-regulated in infiltrative gastric cancer and that miR-145 expression and FSCN1 expression have a strong inverse correlation in infiltrative gastric cancer. Functionally, we demonstrated that miR-145 suppresses cell migration and invasion in gastric cancer primarily by directly targeting FSCN1. Hence, restoring the expression of miR-145 can be explored as an alternative therapeutic strategy against gastric cancer.

## References

[1] Arnold M, Moore SP, Hassler S, Ellison-Loschmann L, Forman D, Bray F. The burden of stomach cancer in indigenous populations: a systematic review and global assessment. Gut. 2014;63:64–71. 10.1136/gutjnl-2013-305033 24153248

[2] Jemal A, Bray F, Center MM, Ferlay J, Ward E, Forman D. Global cancer statistics. CA Cancer J Clin. 2011;61:69–90. 10.3322/caac.20107 21296855

[3] Gupta GP, Massague J. Cancer metastasis: building a framework. Cell. 2006;127:679–95. 17110329 10.1016/j.cell.2006.11.001

[4] Espejo Romero H, Navarrete Siancas J. [Classification of stomach adenocarcinomas]. Revista de gastroenterologia del Peru: organo oficial de la Sociedad de Gastroenterologia del Peru. 2003;23:199–212. 14532921

[5] Ming SC. Gastric carcinoma. A pathobiological classification. Cancer. 1977;39:2475–85. 872047 10.1002/1097-0142(197706)39:6<2475::aid-cncr2820390626>3.0.co;2-l

[6] Bartel DP. MicroRNAs: target recognition and regulatory functions. Cell. 2009;136:215–33. 10.1016/j.cell.2009.01.002 19167326 PMC3794896

[7] Ventura A, Jacks T. MicroRNAs and cancer: short RNAs go a long way. Cell. 2009;136:586–91. 10.1016/j.cell.2009.02.005 19239879 PMC3910108

[8] Zhang BG, Li JF, Yu BQ, Zhu ZG, Liu BY, Yan M. microRNA-21 promotes tumor proliferation and invasion in gastric cancer by targeting PTEN. Oncol Rep. 2012;27:1019–26. 10.3892/or.2012.1645 22267008 PMC3583594

[9] Takagi T, Iio A, Nakagawa Y, Naoe T, Tanigawa N, Akao Y. Decreased expression of microRNA-143 and -145 in human gastric cancers. Oncology. 2009;77:12–21. 10.1159/000218166 19439999

[10] Gao P, Xing AY, Zhou GY, Zhang TG, Zhang JP, Gao C, et al The molecular mechanism of microRNA-145 to suppress invasion-metastasis cascade in gastric cancer. Oncogene. 2013;32:491–501. 10.1038/onc.2012.61 22370644

[11] Kamikihara T, Ishigami S, Arigami T, Matsumoto M, Okumura H, Uchikado Y, et al Clinical implications of N-cadherin expression in gastric cancer. Pathol Int. 2012;62:161–6. 10.1111/j.1440-1827.2011.02774.x 22360503

[12] Vignjevic D, Kojima S, Aratyn Y, Danciu O, Svitkina T, Borisy GG. Role of fascin in filopodial protrusion. J Cell Biol. 2006;174:863–75. 16966425 10.1083/jcb.200603013 PMC2064340

[13] Machesky LM, Li A. Fascin: Invasive filopodia promoting metastasis. Commun Integr Biol. 2010;3:263–70. 20714410 10.4161/cib.3.3.11556 PMC2918773

[14] Hashimoto Y, Kim DJ, Adams JC. The roles of fascins in health and disease. J Pathol. 2011;224:289–300. 10.1002/path.2894 21618240

[15] Hanker LC, Karn T, Holtrich U, Graeser M, Becker S, Reinhard J, et al Prognostic impact of fascin-1 (FSCN1) in epithelial ovarian cancer. Anticancer Res. 2013;33:371–7. 23393326

[16] Takikita M, Hu N, Shou JZ, Giffen C, Wang QH, Wang C, et al Fascin and CK4 as biomarkers for esophageal squamous cell carcinoma. Anticancer Res. 2011;31:945–52. 21498718 PMC3236111

[17] Park SH, Song JY, Kim YK, Heo JH, Kang H, Kim G, et al Fascin1 expression in high-grade serous ovarian carcinoma is a prognostic marker and knockdown of fascin1 suppresses the proliferation of ovarian cancer cells. International journal of oncology. 2014;44:637–46. 10.3892/ijo.2013.2232 24378809 PMC3928475

[18] Fu H, Wen JF, Hu ZL, Luo GQ, Ren HZ. Knockdown of fascin1 expression suppresses the proliferation and metastasis of gastric cancer cells. Pathology. 2009;41:655–60. 10.3109/00313020903273100 20001345

[19] Fuse M, Nohata N, Kojima S, Sakamoto S, Chiyomaru T, Kawakami K, et al Restoration of miR-145 expression suppresses cell proliferation, migration and invasion in prostate cancer by targeting FSCN1. International journal of oncology. 2011;38:1093–101. 10.3892/ijo.2011.919 21258769

[20] Kano M, Seki N, Kikkawa N, Fujimura L, Hoshino I, Akutsu Y, et al miR-145, miR-133a and miR-133b: Tumor-suppressive miRNAs target FSCN1 in esophageal squamous cell carcinoma. Int J Cancer. 2010;127:2804–14. 10.1002/ijc.25284 21351259

[21] Dynoodt P, Speeckaert R, De Wever O, Chevolet I, Brochez L, Lambert J, et al miR-145 overexpression suppresses the migration and invasion of metastatic melanoma cells. International journal of oncology. 2013;42:1443–51. 10.3892/ijo.2013.1823 23404256

[22] Sempere LF, Christensen M, Silahtaroglu A, Bak M, Heath CV, Schwartz G, et al Altered MicroRNA expression confined to specific epithelial cell subpopulations in breast cancer. Cancer Res. 2007;67:11612–20. 18089790 10.1158/0008-5472.CAN-07-5019

[23] Liu X, Sempere LF, Galimberti F, Freemantle SJ, Black C, Dragnev KH, et al Uncovering growth-suppressive MicroRNAs in lung cancer. Clin Cancer Res. 2009;15:1177–83. 10.1158/1078-0432.CCR-08-1355 19228723 PMC3749890

[24] Lu R, Ji Z, Li X, Zhai Q, Zhao C, Jiang Z, et al miR-145 functions as tumor suppressor and targets two oncogenes, ANGPT2 and NEDD9, in renal cell carcinoma. Journal of cancer research and clinical oncology. 2014;140:387–97. 10.1007/s00432-013-1577-z 24384875 PMC11823528

[25] Lauren P. The Two Histological Main Types Of Gastric Carcinoma: Diffuse And So-Called Intestinal-Type Carcinoma. An Attempt at a Histo-Clinical Classification. Acta pathologica et microbiologica Scandinavica. 1965;64:31–49. 14320675 10.1111/apm.1965.64.1.31

[26] Feng Y, Zhu J, Ou C, Deng Z, Chen M, Huang W, et al MicroRNA-145 inhibits tumour growth and metastasis in colorectal cancer by targeting fascin-1. British journal of cancer. 2014;110:2300–9. 10.1038/bjc.2014.122 24642628 PMC4007224

